# Comparative Wound Healing Processes in Plants and Animals: Bioinspired Strategies for Advancing Regenerative Medicine

**DOI:** 10.3390/ijms27135899

**Published:** 2026-06-30

**Authors:** Fatemeh Najafi, Natália Aparecida de Paula, Filipe Rocha Lima, Marcio Fronza, Carem Gledes Vargas Rechia, Marco Andrey Cipriani Frade

**Affiliations:** 1 Translational Laboratory for Skin Studies and Alternative Models, Dermatology Division, Department of Internal Medicine, Ribeirão Preto Medical School, University of São Paulo, Ribeirão Preto 14040-900, SP, Brazil; fnajafi227@gmail.com (F.N.); npbiomed@yahoo.com.br (N.A.d.P.); rfilipelima@gmail.com (F.R.L.); 2 Postgraduate Program in Pharmaceutical Sciences, Natural Products Laboratory, Vila Velha University-UVV, Vila Velha 29102-920, ES, Brazil; marcio.fronza@uvv.br; 3 Department of Biomolecular Sciences, School of Pharmaceutical Sciences of Ribeirão Preto, University of São Paulo, Ribeirão Preto 14040-900, SP, Brazil; cvrechia@fcfrp.usp.br

**Keywords:** wound healing, regenerative medicine, bioelectrical signaling, immune responses, tissue formation, remodeling, plant stem cells, hybrid-wound healing system

## Abstract

Wound healing is a fundamental biological process essential to maintaining structural integrity and survival across both plant and animal life. Despite the profound evolutionary distance separating these kingdoms, wound healing provides one of those momentous occasions when these biological universes collide, revealing significant evolutionary parallels in the core mechanisms of healing, despite clear molecular and physiological differences. However, two challenges have hindered systematic cross-kingdom comparisons. First, unlike animal wound healing, the major phases of plant wound healing have not been organized into a universally accepted classification. Second, no comparative framework exists for systematically comparing wound-healing processes across plant and animal kingdoms. To address these challenges, we developed a comparative classification framework that organizes wound healing into three functional phases: (1) bioelectrical signaling, (2) immune responses, and (3) tissue formation and remodeling. This classification defines the major phases of plant wound healing, while the comparative framework establishes a common basis for systematic cross-kingdom comparison. Through comparative analysis, multiple shared cellular and molecular mechanisms were identified. These findings led to a conceptual model termed the hybrid-wound healing system, integrating plant- and animal-derived regenerative responses and providing a theoretical basis for future bioinspired regenerative strategies. Within this system, living plant stem cells are proposed as central biological components that may potentially act as intelligent pharmaceutical microfactories, releasing bioactive molecules in suitable microenvironments. This approach represents a hypothetical future strategy requiring extensive preclinical validation to strategies based on extracts, conditioned media, extracellular vesicles, or isolated bioactive compounds. Collectively, this descriptive review establishes a conceptual foundation for future investigations in plant biology, wound healing, and regenerative medicine.

## 1. Introduction

Wound healing is a fundamental biological process that restores tissue integrity and function following injury [1]. In animals, wound healing has been extensively investigated because of its critical importance in clinical medicine and tissue regeneration [2]. The healing process is highly dynamic and involves tightly coordinated cellular and molecular interactions that regulate tissue repair and regeneration [3,4]. Although plants and animals diverged early during evolution and developed distinct anatomical and physiological systems, increasing evidence suggests that both kingdoms employ comparable biological strategies to respond to injury and restore tissue integrity [5], despite clear molecular and physiological differences.

Despite growing interest in comparative biology, systematic cross-kingdom analyses of wound-healing processes remain limited. Two major challenges contribute to this limitation. First, unlike animal wound healing, whose major phases are commonly classified into hemostasis, inflammation, proliferation, and remodeling [6,7], plant wound healing lacks a universally accepted classification. Instead, plant wound responses have largely been described through individual processes such as electrical signaling [8], hormone-mediated regenerative signaling [9], tissue regeneration [10], and wound periderm development [11], rather than being integrated into a unified classification of plant wound healing. Second, no comparative framework currently exists for systematically comparing wound-healing processes across plant and animal kingdoms. Consequently, similarities and differences between these systems remain fragmented, limiting the systematic identification of shared regenerative mechanisms and biological principles.

To address these challenges, we developed a comparative classification framework that organizes wound-healing processes in both kingdoms into three broad functional phases: (1) bioelectrical signaling, (2) immune responses, and (3) tissue formation and remodeling. Rather than focusing on kingdom-specific structures or terminology, this framework emphasizes shared biological functions involved in injury perception, defense activation, tissue repair, and regeneration. By establishing a common organizational structure, the framework enables systematic comparison of wound-healing processes across plants and animals and facilitates the identification of conserved cellular and molecular mechanisms that may underlie regenerative responses in evolutionarily distant organisms.

Comparative analysis reveals multiple shared cellular and molecular mechanisms underlying wound healing in plants and animals. In the first phase, both kingdoms initiate rapid wound signaling through glutamate-associated responses [12,13], Ca^2+^ waves [13], reactive oxygen species (ROS) waves [14], hydraulic signals [15], and temperature-related responses [16,17]. Although animals rely on specialized neural tissues for signal transmission, plants coordinate long-distance wound responses through vascular and plasmodesmata-associated bioelectrical communication networks [8,18]. These similarities reflect functional convergence in injury signaling rather than neural similarity between the two kingdoms. In the immune-response phase, both kingdoms activate protective pathways involving lipid-derived signaling molecules, hormonal regulators, and antimicrobial defense mechanisms. Notable parallels include jasmonic acid (JA) and prostaglandin (PG) signaling pathways, which represent analogous lipid-based mechanisms involved in wound and immune responses [19], despite having different precursors and synthesis pathways. In addition, abscisic acid (ABA)-related signaling has been reported in both kingdoms, although its biological roles differ substantially between plants and animals [20]. Melatonin also contributes to oxidative stress management and tissue protection across both kingdoms [21]. Together, these responses support antimicrobial defense, stress adaptation, and wound protection following injury. During tissue formation and remodeling, both kingdoms rely on dynamic extracellular matrix (ECM) or cell-wall remodeling, activation of stem-cell or meristematic populations, vascular regeneration, and conserved regulatory pathways associated with Target of Rapamycin (TOR) and Retinoblastoma-related (RBR) signaling. However, while meristematic populations operate within the constraints of a dynamic cell wall and phytohormone-regulated developmental programs, animal stem cells interact with a flexible ECM and distinct regulatory networks. Although these processes are implemented through distinct developmental programs, they ultimately contribute either to tissue regeneration or to the formation of protective repair structures, including scar tissue in animals and barrier-forming wound periderm in plants [5,10,22,23,24,25]. Collectively, these parallels suggest that injury perception, defense activation, tissue regeneration, and remodeling are organized around shared biological principles, despite the distinct evolutionary histories and structural organization of plants and animals.

The identification of these shared regenerative mechanisms provides an opportunity to move beyond simple comparison toward the development of new conceptual strategies for regenerative medicine. Based on the common biological principles observed across both kingdoms, we propose the Hybrid Wound Healing System as a conceptual model that integrates plant- and animal-derived regenerative responses within a unified framework. Rather than viewing plants and animals as entirely separate biological systems, this concept explores how complementary regenerative features from both kingdoms may inspire innovative approaches to tissue repair and regeneration. Within this conceptual framework, living plant stem cells are proposed as particularly promising biological components because of their regenerative potential, capacity to produce diverse bioactive molecules, and ability to respond dynamically to environmental cues.

Therefore, the objectives of this review are fourfold: (i) to establish a classification of the major phases of plant wound healing, (ii) to develop a comparative framework for systematic cross-kingdom analysis of wound-healing processes, (iii) to identify shared cellular and molecular mechanisms involved in regeneration across plants and animals, and (iv) to propose the Hybrid Wound Healing System as a conceptual model inspired by these shared biological principles. By integrating current knowledge from plant biology, wound healing, and regenerative medicine, this review aims to provide a conceptual foundation for future bioinspired regenerative strategies and interdisciplinary research.

### 1.1. Literature Search Strategy

This study was conducted as a descriptive narrative review aimed at establishing a comparative framework for wound-healing processes in plants and animals, identifying shared regenerative mechanisms across kingdoms, and exploring their potential implications for regenerative medicine. The review further aimed to establish a functional classification framework for plant wound healing and to provide the conceptual foundation for the proposed Hybrid Wound Healing System. A focused literature search was performed using PubMed, Scopus, and Web of Science databases. The literature search was continuously updated throughout the preparation of this review, and the final search update was conducted in May 2026. The included literature ranged from foundational studies published in 1985 to recent articles available up to May 2026. Search terms were used individually and in combination and included: wound healing, plant wound healing, comparative biology, cross-kingdom analysis, bioelectrical signaling, electrical signaling, immune response, tissue regeneration, tissue formation, tissue remodeling, plant stem cells, animal stem cells, callus formation, vascular regeneration, regenerative medicine, biomaterials, biosafety, and wound regeneration. Retrieved publications were screened based on their titles, abstracts, and full texts when necessary. Studies were included if they addressed topics related to wound healing, regeneration, injury signaling, immune responses, stem cell biology, tissue remodeling, vascular regeneration, or comparative biological processes in plants and animals. Both experimental studies and review articles were considered. Studies were excluded if they were unrelated to wound healing or regeneration, focused on non-relevant biological systems, or did not contribute to the objectives of this comparative review. Literature selection was based on scientific relevance, methodological quality, and contribution to the understanding of regenerative mechanisms in plant and animal systems.

### 1.2. Bioelectrical Signaling in Wound Healing

In both plants and animals, wound healing begins with a cascade of rapid and complex signaling events that spread both locally and throughout the organism. These processes involve shifts in membrane potential, ion fluxes, Ca^2+^ signaling, ROS production, temperature fluctuations, and pH alterations, all of which serve as essential early responses to tissue injury [26].

#### 1.2.1. In Plants

Role of Glutamate (GLU) and Ca^2+^ Influx. Following injury, plants initiate rapid cellular responses directly at the wound site. Within seconds to minutes, sudden changes in vascular pressure give rise to hydraulic signals that propagate systemically through the vasculature, altering pressure in distant tissues [15]. This early response is accompanied by phloem-associated signaling and the release of GLU into the apoplast, where it contributes to an increase in apoplastic pH to values above ~6.5 [12], thereby establishing GLU as a key signaling molecule in the activation of defense mechanisms. Within 0.5 to 2 min of injury, membrane depolarization occurs, driven by an influx of H^+^ and Ca^2+^ ions and an efflux of K^+^ and nitrate across the membrane [27].

Generation and Role of ROS, and Electrical Signals. Simultaneously, the production of ROS, such as superoxide anions and H_2_O_2_ is temporarily stimulated in the damaged tissue at the wound site, acting as rapid, long-distance signals [28]. ROS signals generated by mesophyll cells and vascular bundles are coupled with localized temperature increases at the wound site and are further associated with systemic heat signaling throughout the plant [17,29]. Together, ROS and heat signals contribute to the generation of electrical signals that propagate throughout the plant, thereby amplifying the defense response [30]. ROS propagation occurs systemically through ion fluxes and plasmodesmata, contributing to plant-wide bioelectrical signaling. Together with hydraulic signals, Ca^2+^ waves, and membrane depolarization, ROS signaling forms a coordinated, multi-layered systemic signaling network [31]. ROS signaling plays a central role in activating medium- and long-term defense mechanisms essential for systemic protection [32].

#### 1.2.2. In Animals

GLU’s Role in Wound Signaling and Ion Fluxes. Wound healing similarly begins with a cascade of rapid signaling events. Blood leakage at the wound site initiates the release of GLU, a major excitatory neurotransmitter in central nervous systems that also functions as a conserved systemic signaling molecule during wound healing [33]. GLU initiates ion fluxes across the cell membrane, leading to an influx of Ca^2+^ and the activation of intracellular second messenger pathways. This Ca^2+^ pulse represents the onset of wound signaling, facilitating the rapid upregulation of immediate early genes that drive the healing response [12]. At the wound margin, intracellular Ca^2+^ concentrations rapidly rise, peaking within six seconds in vascular and epidermal cells before propagating outward as a Ca^2+^ wave [34,35]. The Ca^2+^ wave is accompanied by an increase in pH; while the stratum corneum normally maintains an acidic pH (4.1–5.8), the wound site initially becomes alkaline before gradually returning to an acidic state as healing progresses [36].

ROS and Electric Fields in Early Wound Responses. Simultaneously, ROS, particularly H_2_O_2_, is produced at the wound site, acting as a long-range messenger that regulates various aspects of the healing process. Within minutes of injury, endogenous electric fields acting in concert with Ca^2+^ waves, cellular depolarization, and H_2_O_2_ signaling, recruit inflammatory cells to the wound site [37,38]. These electric fields, together with ROS and ion fluxes, form an integrated signaling network that coordinates early wound responses and establishes the framework for subsequent phases of healing. Wound hypoxia is typically the highest at the wound center, which can impair immune cell function and delay healing. Adequate oxygenation is necessary for processes such as collagen deposition and angiogenesis [39]. Additionally, studies have shown that a skin temperature of 33 °C is optimal for immune cell activity during wound healing [16]. Wound pH also plays a regulatory role, with a more neutral pH favoring healing [36].

#### 1.2.3. Comparative Aspects

Plants and animals share fundamental principles in their signaling mechanisms, utilizing neurotransmitter-like substances and bioelectric processes to regulate physiological responses. In animals, rapid signal transmission is mediated by specialized neural tissues; in contrast, plants lack discrete neurons and instead utilize integrated vascular-based communication networks that enable long-distance signal propagation. Within these networks, phloem-associated cells, including companion cells, play a key role in coordinating systemic signaling across tissues [18]. Recent advances in plant neurobiology and electrophysiology have revealed notable conceptual and mechanistic parallels with signaling processes observed in animals. However, these similarities are considered functionally analogous rather than indicative of neuronal homology, as plants lack specialized neural tissues and nervous systems comparable to those of animals [40]. Across both kingdoms, ion fluxes including Ca^2+^, K^+^, and Cl^−^ underpin the generation and propagation of electrical signals, such as action potential-like events. In addition, several compounds traditionally associated with animal neurotransmission, including acetylcholine, dopamine, serotonin, and melatonin, are also present in plants, where they contribute to development, stress responses, and intercellular communication [41]. Together, these observations support the view that plants and animals have evolved distinct structural solutions to achieve partially convergent signaling functions, reflecting shared biophysical principles rather than true neural homology.

GLU and Ion Fluxes in Plants and Animals. In both plants and animals, GLU is essential for wound signaling, though it operates through distinct receptor systems in each kingdom. In plants, glutamate receptor-like (GLR) ion channels act as sensors for injury, while in animals, GLU functions as a neurotransmitter involved in initiating wound signaling [13]. Ca^2+^, a ubiquitous second messenger in both, generates Ca^2+^ signatures that are integral to distinguishing between stimuli and regulating downstream responses. The fluctuating pH at the wound site, driven by ion fluxes and Ca^2+^ waves, further modulates metabolic activity during repair, highlighting a shared regulatory mechanism [42]. While long-range calcium propagation is well documented in plant vascular tissues, calcium waves in animal epithelial tissues are generally more localized and are primarily coordinated through gap junctions and purinergic signaling.

**Figure 1:**
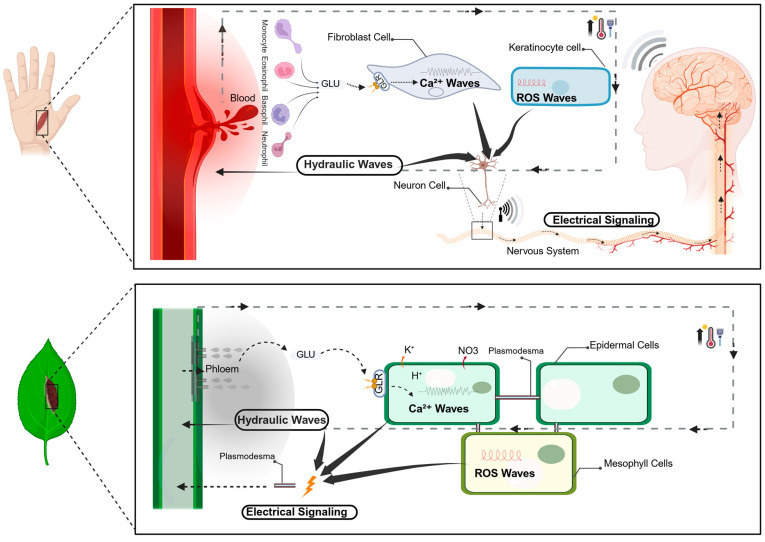
Comparative mechanisms of wound-induced bioelectrical signaling in animals and plants. The schematic illustrates key similarities and differences in systemic wound signaling pathways. In animals, tissue injury triggers GLU release, immune-cell recruitment, Ca^2+^ and ROS waves, and neuronal electrical signaling that coordinate local and systemic responses. In plants, wounding induces phloem secretion and GLU release, leading to membrane depolarization, Ca^2+^, ROS, and hydraulic waves that propagate through vascular tissues and surrounding cells. Although plants lack a nervous system, systemic signaling is achieved through ion fluxes, ROS propagation, and cell-to-cell communication via plasmodesmata. Together, these mechanisms highlight conserved roles of Ca^2+^, ROS, and bioelectrical signaling in coordinating wound responses across kingdomsCreated in BioRender. Lima, F. (2026) https://BioRender.com/oxa7whl, accessed on 8 May 2026.

ROS and Electric Fields in Early Responses in Plants and Animals. ROS are also critical in both systems, acting as a microbicidal, stress signals and long-range messengers. While in animals, ROS modulate immune cell recruitment and inflammation, in plants, ROS propagate through a wave system to alert distant tissues of damage. This parallel signaling framework, combining electric fields, ROS, and ion fluxes, creates an integrated response to injury in both systems, initiating the early phases of healing [14] (Table 1 and Figure 1).

### 1.3. Immune Responses in Wound Healing

Wound healing involves not only rapid signaling of tissue damage but also activation of immune mechanisms that support healing and prevent infection. Although plants and animals differ substantially in their immune systems, both utilize complex and coordinated responses to injury. In both kingdoms, early defense responses are closely associated with hormonal signaling, activation of defense-related genes, and production of immune-related compounds [43].

#### 1.3.1. In Plants

Hormonal and Defense Responses. Plants exhibit a complex immune response that involves both local and systemic signaling pathways, primarily regulated by phytohormones. Among these regulators, JA, ABA, and melatonin play central roles in mediating wound responses [19]. JA is a key regulator of wound signaling. Upon tissue damage, JA biosynthesis is rapidly induced, leading to a transient wave of accumulation within 10 min to 2 h that typically subsides by 4 h [44]. These JA-mediated signals function not only locally at the wound site but also systemically, extending to distal organs via vascular connections [45]. Moreover, the initial JA peak promotes auxin production, thereby facilitating tissue regeneration [46]. ABA, another stress-responsive hormone, contributes to the regulation of programmed cell death at the wound site, helping to limit tissue damage and reduced risk of pathogen invasion. In addition, ABA promotes defense-associated responses, including the deposition of callose, suberin, and lignin, which reinforce physical barriers against further injury or infection [47,48]. Melatonin has emerged as an important regulator in plant immune responses, acting alongside signaling molecules such as nitric oxide (NO) and phytohormones including salicylic acid and JA [49]. It participates in phytohormone-mediated signal transduction pathways [21]. Furthermore, melatonin acts as a potent antioxidant, directly scavenging reactive species such as ROS, reactive nitrogen species (RNS), and NO, owing to its amphipathic nature which enables it to cross cellular membranes and distribute across multiple organelles [50].

**Figure 2:**
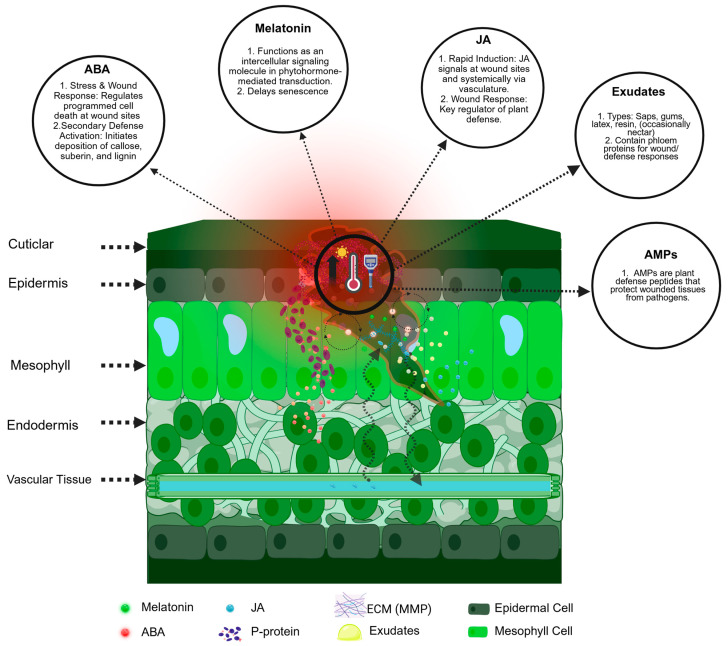
Immune responses in plant wound healing. Plants activate a complex immune response upon wounding, integrating phytohormones and structural defenses. JA initiates rapid wound signaling, while ABA regulates programmed cell death and secondary defenses. Melatonin modulates immune responses and neutralizes reactive species. Plant exudates, including latex and resin, contribute to antimicrobial defense. Latex coagulates to seal wounds and block pathogens, while resins provide antiseptic protection, maintaining wound integrity. AMPs accumulate at wound sites and outer tissue layers, providing rapid antimicrobial protection against invading pathogens. Created in BioRender. Lima, F. (2026) https://BioRender.com/ul6o0no, accessed on 8 May 2026.

Wound Exudates. Plant exudates, including sap, gum, latex, and resin, are released at wound sites and contribute to defense through antimicrobial compounds such as alkaloids, terpenoids, phenolics, and proteases [51]. Latex, secreted by specialized laticifer cells, rapidly coagulates upon air exposure, sealing wounds and limiting microbial colonization [52]. In parallel, antimicrobial peptides (AMPs), present in most plant species, play a key role in wound defense by protecting injured tissues from pathogen invasion. Major AMP families include thionins, plant defensins, and lipid-transfer proteins, which are enriched in outer plant tissues. Thionins occur both intra- and extracellularly, whereas defensins and lipid-transfer proteins are primarily extracellular. Pathogen infection typically induces strong upregulation of genes encoding these peptides [53] (Table 1 and Figure 2).

#### 1.3.2. In Animals

Most animal species possess a developed immune system that has evolved to detect and clear pathogens whenever the protective barrier is breached and infection ensues [54].

Hormonal and Defense Responses. The immune response initiates within minutes of injury [55]. During this period, pre-synthesized mediators stored in intracellular granules, growth factors (GFs), cytokines, and chemokines initiate an immune response, activating resident cells and recruiting neutrophils, macrophages, and lymphocytes to the injury site [56,57]. Some immune cells, such as neutrophils and macrophages, are rapidly activated through interactions with resident tissue cells, particularly keratinocytes, as well as with platelets and wound-derived molecular signals [58]. The inflammatory response in wound healing is tightly regulated. Tumor Necrosis Factor (TNF), Interleukin-1 (IL-1), Interleukin-6 (IL-6), Interleukin-8 (IL-8), Platelet-Derived Growth Factor (PDGF), Transforming Growth Factor-beta (TGF-β), Vascular Endothelial Growth Factor (VEGF), Fibroblast Growth Factor (FGF), and Interleukin-10 (IL-10) form the essential molecular profile that coordinates and drives early wound healing. These mediators orchestrate key cellular processes, including migration, proliferation, and remodeling, with dynamic changes in this molecular profile occurring throughout the healing process [59]. GFs enhance cellular communication, stimulate stem cell activity, and counteract cellular senescence [60].

Prostaglandin E2 (PGE2) plays a central role in vasodilation and inflammation and regulates immune responses throughout the wound-healing process [61]. Leukocytes and mesenchymal stem cells (MSCs) produce ABA, a conserved hormone in plants and humans, with immunoregulatory roles in mammals, influencing immune cell activity, aiding in the stress response during wound healing, and promoting the expansion of MSCs and colon stem cells [20]. Additionally, melatonin contributes to wound healing through its antioxidant and anti-inflammatory effects, reducing oxidative stress and regulating inflammasome activation [51].

**Figure 3:**
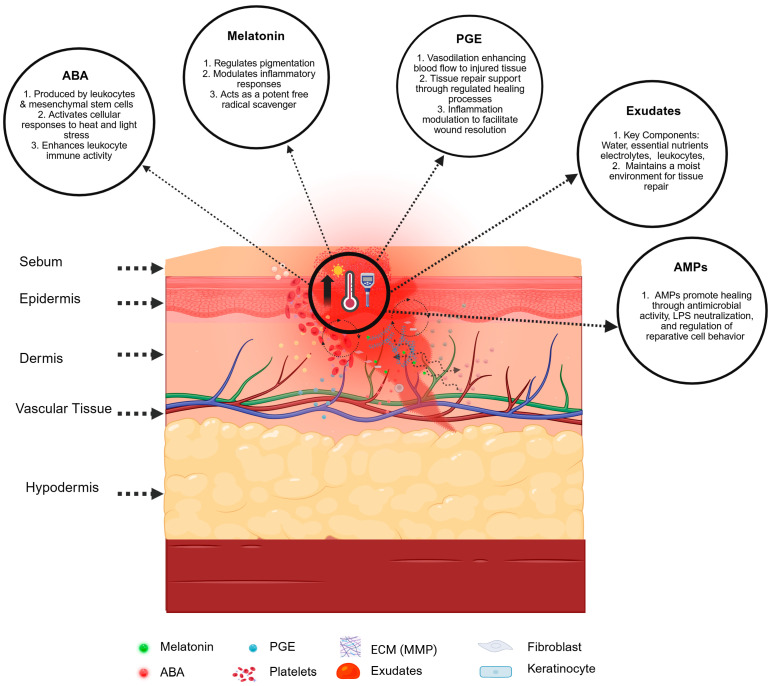
Immune responses in animal wound healing. After injury, the immune response is rapidly activated through the release of lipid mediators, GFs, cytokines, and chemokines, facilitating the recruitment of neutrophils, macrophages, and lymphocytes to the wound site. PGE2 and other mediators modulate vasodilation and inflammation across all phases of wound healing. ABA contributes to immune regulation and stem cell activation, while melatonin mitigates oxidative stress and inflammation. Wound exudates maintain a moist, bioactive environment and contribute to innate immune defense, supporting cell migration, proliferation, and tissue regeneration. AMPs promote healing through antimicrobial activity, LPS neutralization, and regulation of reparative cell behavior. Created in BioRender. Lima, F. (2026) https://BioRender.com/8fdsjc2, accessed on 8 May 2026.

Wound Exudates. Wound exudate, also known as exudate, exhibits inherent AMPs and plays a critical role in the innate immune response by protecting compromised tissues from infection. Wound exudate acts as a hydrated reservoir containing nutrients, electrolytes, leukocytes, inflammatory mediators, GFs, and proteolytic enzymes, thereby maintaining a moist wound environment while delivering bioactive signals essential for effective wound healing [62]. Among these components, AMPs act as key effectors of innate immunity, contributing to host defense through antimicrobial activity, neutralization of microbial antigens that induce tissue damage and inflammation such as lipopolysaccharides (LPS), and modulation of cell migration and proliferation during wound healing [52]. Human β-defensin 2 (hBD-2) accelerates wound healing by stimulating keratinocyte migration through the activation of the Epidermal Growth Factor Receptor (EGFR), promoting skin re-epithelialization [4] (Table 1 and Figure 3).

#### 1.3.3. Comparative Aspects

In both animals and plants, the immune response plays a crucial role in wound healing. In plants, the immune system is highly sophisticated, relying on a vast array of immune receptors to detect a wide range of pathogens, which compensates for their lack of humoral immunity [63].

Hormonal and Defense Responses in Plants and Animals. One significant cross-kingdom similarity lies in the role of phytohormones produced by plants but with biological effects in animals. It has been demonstrated that humans or human cell cultures can also create similar signal molecules naturally [53]. Plant cells can synthesize hormones locally, whereas animal hormones are typically produced in specialized organs and transported via the bloodstream [64].

JA vs. PGs. In animals, PGs regulate inflammation during wound healing. Produced from membrane lipids via phospholipase A_2_, they generate substrates for lipoxygenases, paralleling JA signaling in plants [19]. These pathways reflect an analogous lipid-based mechanism controlling immune and healing responses, despite significant molecular and biosynthetic differences.

ABA functions as a universal signaling molecule in both plants and animals. In animals, ABA activates a signaling pathway that raises intracellular Ca^2+^ concentrations, functioning in a way comparable to its role in plants, where it modulates stress responses, even in the evident differences between the Ca^2+^ wave in plants and the processes orchestrated via Ca channels and cell junctions in animals. The ABA pathway in animals involves cyclic ADP ribose and is linked to immune modulation and cellular regeneration [20].

Melatonin, an indoleamine found in both plants and animals, plays an important role in oxidative stress management and tissue protection across species. In animals, melatonin acts as an antioxidant and modulates immune responses, whereas in plants, it helps protect cell membranes from oxidative damage [21].

Plant wound exudates, such as latex and resin, and animal wound exudates both contribute to wound protection and defense against microbial invasion. Although their composition, origin, and biological functions differ substantially between kingdoms, both participate in maintaining a favorable wound environment during the healing process. These factors, including pH levels, exudate composition, and wound temperature act as valuable indicators of the healing process [65]. While plant exudates rely on static chemical seals, animal exudate is a dynamic vascular response. However, tight control of this fluid volume is essential; while adequate exudate delivers critical immune cells and antimicrobial factors, excessive or stagnant accumulation disrupts cellular migration, promotes chronic inflammation, and creates a breeding ground for bacterial infections.

AMPs are small, low-molecular-weight proteins with broad-spectrum activity against Pathogen-Associated Molecular Patterns (PAMPs) of bacteria, viruses, and fungi, and activated during inflammation via Damage-Associated Molecular Patterns (DAMPs) [66]. In plants, elicitor peptides such as regeneration factor 1 (REF1) coordinate wound-induced defense and regeneration through cellular reprogramming pathways [67]. In animals, the cathelicidin LL37 exhibits antimicrobial and immunomodulatory functions, suppressing inflammation and promoting angiogenesis [68]. While animal wounds are sealed by fibrin clots, plants form a callose-based barrier at wound margins, with locally produced AMPs providing additional protection against infection [5] (Table 1).

### 1.4. Tissue Formation and Remodeling in Wound Healing

Tissue formation and remodeling represent fundamental processes in wound healing, driving wound closure through coordinated cellular activation, matrix deposition, and restoration of local structural continuity. Remodeling subsequently reorganizes and matures the newly formed tissue, determining whether healing results in regeneration (callus in plants; blastema in animals) or repair with scar formation (suberization/lignification in plants; fibrosis in animals).

#### 1.4.1. In Plants

In plants, wound responses such as callus formation, suberization, cell wall remodeling, and regeneration have been described in a fragmented way. Here, we integrate these processes into a unified wound-healing framework to enable cross-kingdom comparison with animal systems.

Tissue formation in plants. The plant ECM, which encompasses the cell wall and its associated extracellular proteins, signaling molecules, and structural components, serves as a dynamic interface involved in growth, development, responses to biotic stress, and wound-induced processes [22,69]. Although structurally distinct from the ECM of animals, the plant ECM functions as a specialized interface for signal transduction, cellular communication, and tissue responses during wound healing. Remodeling of this extracellular environment is mediated in part by plant matrix metalloproteinases (MMPs), zinc-dependent endopeptidases that modify extracellular and cell wall proteins during tissue repair and regeneration. Through extracellular remodeling, plant MMPs implicated in tissue reorganization, regeneration, and wound-healing processes following injury [24].

Remodeling In Plants. Wound remodeling involves regulated structural and functional reorganization following tissue formation in response to injury signals. This process spans multiple scales, from chromatin dynamics to cell wall remodeling and tissue architecture changes. During regeneration, the cell wall functions as a dynamic interface that integrates signals to control cell fate. Selective degradation and deposition of wall components provide the plasticity required for cellular reprogramming [70]. Plant regeneration refers to the ability of differentiated cells or tissues to reprogram their developmental fate, enabling repair of damaged structures or the formation of new organs [69]. After injury, regeneration may range from localized cell proliferation to full restoration of the damaged tissue or organ, depending on injury severity and developmental stage [10]. Although the stages of plant wound healing remain incompletely defined, evidence supports a distinct remodeling phase in which healing proceeds toward either regeneration (callus formation and organogenesis) or repair through scar formation.

Callus Formation in Plants. Calli are heterogeneous cell populations organized into three transcriptionally distinct layers: outer, middle, and inner [71]. A callus typically represents a disorganized tissue proliferation that develops at wound stumps and in response to specific pathogens [72]. Callus formation represents a critical phase in plant regeneration, facilitating wound healing and the restoration of damaged tissues. This process, characterized by the accumulation of undifferentiated cells at the wound site, provides the structural and cellular framework necessary for subsequent tissue regeneration [73]. Cytokinin signaling promotes extensive cell proliferation in the damaged tissue [74]. Cytokinin plays a key role in reactivating cell division in mature plant tissues, generating undifferentiated cells capable of forming new tissues. This cellular reprogramming enables plants to regenerate damaged or lost organs, particularly in long-lived species such as trees, where regeneration may take several years [75].

Plant Stem Cells. These cells possess specialized regenerative properties that support both development and regeneration. A key feature is their ability to form de novo during plant development and in response to tissue damage. These cells maintain pluripotency and continuously give rise to new organs, contributing to long-term plant survival [74]. Plant stem cells persist and respond to external signals, thereby supporting regeneration following mechanical or pathogen-induced damage, in part through efficient antiviral defense mechanisms [76]. The regenerative capacity of plant stem cells is regulated by plant hormones including auxin, cytokinin, gibberellins, and JA [77]. Auxin and cytokinin are central regulators of stem cell activity. Cytokinin promotes cell proliferation by stimulating cell cycle progression, while auxin facilitates cell differentiation, crucial for the repair of damaged tissues [78]. In response to wounding, JA signaling enhances auxin production, promoting stem cell activation and tissue regeneration [79]. Stem cells located in shoot and root meristems maintain pools of actively dividing cells, and their activity is influenced by environmental conditions such as biotic and abiotic stresses and nutrient availability [80].

Plant Vascularization. Vascular regeneration in plants represents a fundamental and dynamic aspect of post-injury recovery, enabling the restoration of long-distance transport networks that maintain physiological continuity and structural integrity [81]. Following mechanical injury, such as bark removal, vascular tissues, including the cambium and phloem, are often disrupted or lost. In response, plants activate coordinated regenerative responses involving callus proliferation and/or dedifferentiation of existing cells, depending on the extent and nature of the damage [72]. In cases where small portions of bark are removed, regeneration proceeds through the formation of a wound periderm and the emergence of a wound cambium associated with proliferating callus tissue at the trunk surface [82]. This newly established cambium subsequently gives rise to functional phloem, re-establishing vascular flow. However, after extensive girdling, a different pathway is activated. In some species subjected to extensive girdling, both sieve elements (SEs) and the regenerated cambium have been shown to derive from differentiating xylem cells [83]. This process generally involves three principal phases: callus formation and dedifferentiation, emergence of SEs, and subsequent formation of the wound cambium, ultimately in bark regeneration within a month in genus such as *Populus* and *Eucommia* [72]. Auxin plays a key role in vascular regeneration, as its polar transport and localized biosynthesis help re-establish vascular identity at wound sites [83]. The canalization model explains how auxin movement through damaged tissues reinforces its own transport pathways, directing the differentiation of vascular precursors into organized strands [84]. Experimental injury to Arabidopsis leaf midveins has shown that plants can regenerate both partially and fully severed vascular strands. In approximately 80% of cases, regeneration occurs through reconnection of the original strands or through the formation of new lateral connections that bypass the damaged region [25]. At the systemic level, auxin flux and polarized efflux carriers operate within a regulatory feedback loop that governs vascular differentiation and other developmental processes such as phyllotaxis and branching [85].

Plant Scar Formation. In plant systems, structures often described as “scar” represent a specialized wound-induced repair strategy in which tissue integrity is restored through the establishment of a protective barrier. This response is triggered by mechanical injury, pathogen invasion, or developmental processes such as abscission, and represents an important defense mechanism of plant defense aimed at preventing dehydration and microbial entry [86]. Immediately after injury, exposed outer cells rapidly desiccate and die, forming a transient interface with the environment. In contrast, suberization and lignification processes are not localized at the exposed wound surface but instead occur several cell layers inward, where underlying parenchyma cells activate defense pathways and generate a chemically reinforced boundary that isolates damaged tissues [87,88].

This early sealing phase is followed by the formation of a wound periderm, a secondary protective tissue that replaces the damaged epidermis. The process is driven by the differentiation of the phellogen (cork cambium) from dedifferentiated cells beneath the wound site. The phellogen produces suberized phellem outward and phelloderm inward, forming a multilayered barrier system. The protective function of this structure is primarily conferred by suberin deposition, a complex lipophilic biopolymer composed of polyaliphatic and polyaromatic domains that establish an effective barrier against water loss and pathogen invasion [89]. In fruit systems such as tomato, wound-induced suberization of stem scar tissue has been shown to significantly reduce transpiration and susceptibility to microbial infection, highlighting its functional importance in post-harvest protection [86]. Lignification acts as an early complementary process in scar formation, reinforcing cell walls and contributing to the establishment of a boundary zone that precedes full periderm differentiation. Together, lignin and suberin form a coordinated ligno-suberized interface that provides both mechanical strength and hydrophobic sealing, ensuring efficient wound closure and long-term protection. At the physiological level, these structural changes are initiated by rapid wound signaling events, including ROS production, ion fluxes, and hormonal regulation, particularly by ABA, which plays a central role in activating suberin biosynthesis and wound-responsive gene expression [19,90].

#### 1.4.2. In Animals

Tissue formation in animals. This process is mediated by coordinated activation of keratinocytes and fibroblasts alongside dynamic ECM deposition and MMPs-dependent matrix turnover, which together facilitate cell migration, mechanical integration, and wound closure [91].

ECM. Macromolecules such as collagen, elastin, fibronectin, vitronectin, integrins, and laminins constitute the ECM, a three-dimensional structure that provides essential structural and biochemical support to surrounding cells. The ECM is essential for repair of epithelial and connective tissues [92]. During the remodeling phase, the balance between matrix MMPs and tissue inhibitors of metalloproteinases (TIMPs) regulate matrix degradation, while TGF-β coordinate tissue contraction by myofibroblasts and the replacement of type III collagen with type I to restore tensile strength. At the wound margins, keratinocytes release matrix MMPs, including MMP-1, which degrade the ECM and reduce keratinocyte adhesion, thereby promoting cell migration and facilitating wound healing [93].

Remodeling In Animals. This phase determines whether wound healing proceeds toward regeneration or follows a repair pathway associated with scar formation. Regenerative remodeling is characterized by the restoration of tissue structure and function without scar formation and may occur through restoration (“putting together what is broken”) or reconstruction (“replacing and rebuilding what is torn down”) [94]. This process is typically seen in tissues with high proliferative capacity, such as the epidermis, gastrointestinal (GI) tractepithelium, and hematopoietic systems. In the case of stable tissues like the liver and kidney, compensatory growth rather than true regeneration can occur. Conversely, repair relies on collagen deposition to form a scar, particularly when regenerative potential is limited, or the injury is extensive. Thus, while superficial skin wounds may heal through regeneration, deeper or more severe injuries frequently result in scar formation, permanently altering the tissue’s original structure [95].

Blastema Formation. Regeneration occurs in two principal phases: (1) blastema formation, resembling an early embryonic limb bud, and (2) redevelopment, involving growth and redifferentiation. The blastema arises as undifferentiated cells accumulate beneath the thickened distal wound epidermis, known as the apical epidermal cap (AEC). This reverse developmental process is driven partly by local cellular dedifferentiation and partly by contributions from muscle stem cells [96]. Blastema formation is a defining feature of regeneration in many animal species, particularly in highly regenerative vertebrates and invertebrates. The blastema is defined as a mass of undifferentiated cells with mitotic capacity, crucial for the regrowth of lost body parts. While the initial response to injury in virtually all multicellular animals involves rapid wound closure, the formation of a blastema is key to regeneration in animals such as amphibians and invertebrates. These animals can regenerate complex structures, such as limbs or tails, due to the presence of this cellular mass at the injury site [97]. Blastemal cells arise from various sources and mechanisms, including the dedifferentiation of mature cells, transdifferentiation, and the migration of established adult stem cell niches [98]. The blastema is a heterogeneous tissue comprising both multipotent and lineage-restricted progenitor cells, some originating from stem cell activation and others potentially arising through dedifferentiation [99]. Macrophages are important regulators of blastema formation. In mammals, they facilitate wound repair through bactericidal and phagocytic functions, as well as by secreting GFs and cytokines that regulate inflammation and promote fibroblast-driven tissue repair. Studies further highlight the significance of the immune system, particularly macrophages, in the formation of blastema during urodele limb regeneration [96]. Blastema formation is regulated by retinoic acid and various GFs signals, with FGF and WNT/β-catenin pathways playing particularly important roles in driving cell cycle re-entry [100]. This collection of relatively undifferentiated progenitor cells proliferates and repatterns to form the internal tissues of the regenerating body part, including bones, nerves, and muscles [99]. Interestingly, mammals, while generally considered to have poor regenerative capacity, do exhibit blastema formation in specific cases, such as digit-tip regeneration in animals and rodents. After digit-tip amputation, an accumulation of mesenchymal progenitor cells forms a blastema, coordinating the regeneration of various tissue types, including bone and connective tissue. Recent studies have highlighted the role of the nail organ in promoting blastema formation and digit-tip regeneration, suggesting that specific tissue interactions are critical to initiating and sustaining regeneration pathways in mammals [101]. The blastema originates from proliferating mesenchymal-like cells that accumulate above the stump, forming loose connective tissue covered by regenerating or wound epidermis. Progressive proliferation and differentiation of these cells ultimately create the new tail in lizards. In contrast, following a pronounced and prolonged inflammatory response in the limb, no blastema develops, and the connective tissue instead forms scar tissue, mirroring mammalian wound healing. Consequently, lizard regeneration, particularly when compared to amphibian models, offers a valuable perspective on the constraints of tissue regeneration in amniotes, including mammals. When regeneration in lizards is halted by wounding or cauterizing the regenerating tail, the resulting cicatrizing outgrowth provides an opportunity to investigate how scar formation alters the expression of specific molecules, including GFs. This process produces a dense, irregular connective tissue that supplants the normal mesenchymal tissue characteristic of the blastema [102].

Animal Stem Cells. These cells contribute significantly to wound healing by providing undifferentiated precursor cells that can specialize in various tissue types depending on their location. These cells exhibit high self-renewal capacity and resistance to aging, making them important for sustaining tissue homeostasis and facilitating regenerative processes [103]. Specifically, during the final phase of wound healing, known as re-epithelialization, basal keratinocytes and regenerative epidermal stem cells (EPSCs) are activated to restore skin integrity. These multipotent EPSCs originate from diverse sources, including hair follicles, sebaceous glands, and the interfollicular epidermis [60]. Animal stem cells also exhibit antiviral defense mechanisms. For instance, human stem cells utilize antiviral Dicer (AviD) to defend against ribonucleic acid (RNA) viruses [104]. In animals, the stem cell niche, a highly specialized and dynamic microenvironment, is essential for maintaining stemness and regenerative capacity, ensuring that stem cells can respond effectively to injury or disease [105]. GFs are proteins that are essential for regulating stem cell behavior and tissue regeneration, acting both locally and systemically to influence a range of cellular activities, including division, differentiation, and survival. In the context of stem cell-based regeneration, GFs are essential for processes such as blastema formation, as well as the dedifferentiation and redifferentiation of cells, which are key to the replacement of lost or damaged tissues, such as limbs. The effects of GFs can be either pro-regenerative or pro-fibrotic, depending on the specific context and the nature of the damage to the organ, underscoring their importance in determining regenerative outcomes [106].

Animal Vascularization. During skin wound healing, formation of new blood vessels is essential [107]. Vasculogenesis, although mainly an embryonic process, can also occur postnatally under regenerative or pathological conditions. In this context, bone marrow-derived endothelial progenitor cells (EPCs) are mobilized to the injury site, where they proliferate and differentiate into mature endothelial cells (ECs), forming new vascular structures in previously avascular regions [108]. In contrast, angiogenesis the sprouting of new vessels from pre-existing ones is the predominant mechanism during adult wound healing, driving granulation tissue formation and supporting ECM deposition and tissue regeneration [109,110]. The angiogenic response is rapid, generating a dense but initially immature vascular network that is later refined through pruning and stabilization [111].

Animal Scar Formation. In mammals, scar formation is a common consequence of tissue repair. During wound healing, exudates provide GFs, nutrients, and immune cells that support tissue repair while maintaining a moist environment [112,113]. The remodeling phase typically begins about one week after injury and may persist for several months. Excessive or prolonged inflammatory and proliferative responses, such as those associated with infection or burns, increase the likelihood of fibrotic scar formation [114]. Reduced infiltration of inflammatory cells, including mast cells, neutrophils, and macrophages, has been associated with decreased scar formation, highlighting the importance of controlled immune responses during healing [115]. In mammals, scar formation is largely driven by collagen accumulation mediated by TGF-β signaling, which regulates ECM organization and promotes fibroblast proliferation and migration. However, notable exceptions include scar-free healing in pre-third-trimester human fetal skin, the human oral cavity under physiological conditions, and the African spiny mouse, which can regenerate hair follicles and sweat glands after extensive skin injury. Experimental studies further show that repeated limb amputation impairs regenerative capacity and promotes scar-like tissue formation, accompanied by gene expression profiles resembling fibrotic healing. These findings demonstrate the role of reduced fibrosis and a permissive wound epidermis for enabling blastema formation and successful regeneration [99].

#### 1.4.3. Comparative Aspects

##### Tissue Formation

Tissue formation during wound healing in both plants and animals depends on early cellular coordination within a dynamically regulated ECM environment. Rapid ECM-associated responses are essential for early wound sealing, limiting fluid loss and stabilizing the injured site. In plants, vascular damage triggers the release of P-proteins that form reversible disulfide-linked polymers, sealing disrupted phloem elements and preventing sap leakage, functionally analogous to fibrin-based clot formation in animals [116]. Despite structural differences between plant and animal ECM, both systems play fundamental roles in regulating growth, development, and stress responses [117]. In animals, matrix MMPs are key regulators of ECM remodeling, facilitating tissue restructuring and the release of bioactive molecules that influence cell migration and repair [118]. Plant MMPs share conserved structural features with mammalian MMPs, including the cysteine-switch motif and zinc-dependent catalytic domains. Although less extensively characterized, plant MMPs have been implicated in ECM remodeling during growth, development, and responses to biotic and abiotic stresses [24,119]. Despite distinct tissue organization and physiological mechanisms across kingdoms, the remodeling phase follows parallel tissue-stabilizing principles in both plants and animals.

##### Remodeling

Remodeling operates across both plant and animal kingdoms as a shared biological framework through which injured tissues undergo coordinated structural and functional reorganization following damage. This phase ultimately determines healing outcomes by directing tissue responses toward one of two principal trajectories: regeneration or repair. Regenerative remodeling restores tissue structure and function through processes such as vascular regeneration, blastema formation in animals, and callus formation in plants. In contrast, repair-oriented remodeling leads to scar formation, which re-establishes barrier integrity and mechanical stability but does not fully restore the original tissue architecture or physiological function [114]. Stem cells contribute significantly to regenerative remodeling in both kingdoms. In animals, tissue-resident and recruited stem or progenitor cells contribute to tissue reconstruction, whereas in plants, regeneration is supported by totipotent cells within meristems or by somatic cells that undergo dedifferentiation at the wound site [23]. Vascular regeneration also represents a key component of remodeling, although the underlying developmental pathways differ. In animals, angiogenesis and vasculogenesis restore perfusion and nutrient delivery, whereas in plants vascular continuity is re-established through cellular reprogramming processes that regenerate xylem and phloem tissues [120,121]. Regenerative capacity varies considerably across organisms. Plants can regenerate entire organs or even whole individuals from cuttings through callus-mediated cellular reprogramming, although localized tissue replacement may be limited by regulatory feedback associated with cell wall biogenesis at wound sites [122]. Regenerative competence also varies widely among plant species, with some woody species such as *Taxus chinensis*, *Metasequoia glyptostroboides*, and *Ginkgo biloba* showing relatively limited regeneration compared with highly regenerative species including *Ficus virens* and marine algae such as *Laminaria japonica* and *Undaria pinnatifida* [120]. Among animals, regenerative potential is similarly heterogeneous. Planarians can reconstruct entire body structures from small tissue fragments (Morgan 1898), while salamanders demonstrate blastema-mediated limb regeneration and scar-free healing responses [121]. In contrast, vertebrates such as mice exhibit more restricted regenerative capacity [23]. In humans, regeneration is largely limited to tissues with high cellular turnover, including skin, intestinal epithelium, and bone, while organs such as the heart and stomach display poor regenerative potential, whereas the liver retains substantial regenerative capacity [123,124].

In animals, regeneration involves the complete replacement of damaged tissue with new tissue that does not form a scar and may occur via restoration (“putting together what is broken”) or reconstruction (“replacing and rebuilding what is torn down”) [94]. Generally, remodeling involves activation of stem and progenitor cells, angiogenesis, and the reestablishment of tissue-specific structure [125]. Generally, tissue remodeling involves activation of stem and progenitor cells, angiogenesis, ECM remodeling, and the progressive restoration of tissue-specific architecture [126]. This phase forms the foundation for regeneration in tissues with high proliferative capacity such as skin and intestinal epithelium or for scar formation in tissues with limited regenerative potential. This process is regulated by fibroblast activity, TGF-β signaling, and immune cell modulation, particularly by macrophages, which influence whether regeneration or fibrosis ensues [99].

In plants, the response to injury varies depending on its type and severity, resulting in either the formation of protective barriers or regenerative callus tissue [71]. For example, in certain tissues such as fruit epidermis or leaf margins, injury leads to localized suberization and the formation of protective barriers that prevent desiccation and microbial invasion [86]. In contrast, deeper or more severe injuries, particularly in stems and roots, often induce callus formation, a regenerative response involving the accumulation of pluripotent or dedifferentiated cells capable of reconstituting damaged tissue [127].

**Figure 4:**
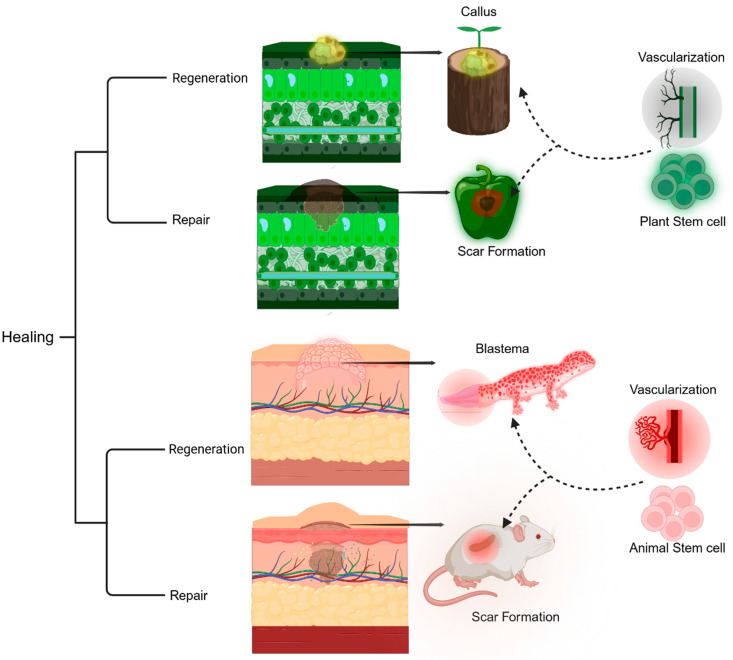
Tissue formation and remodeling in wound healing (plants versus animals). Tissue formation (like ECM deposition) enables wound closure, after which remodeling determines whether healing proceeds toward regeneration (callus in plants; blastema in animals) or repair with scar formation (suberization/lignification/periderm in plants; fibrosis in animals). Created in BioRender. Lima, F. (2026) https://BioRender.com/s9j1w5w, accessed on 17 June 2026.

Callus Formation vs. Blastema Formation. The regeneration pathways of callus formation in plants and blastema formation in animals illustrate parallel strategies adapted to distinct biological organizations and phylogenetically distant lineages. Divergent developmental programs and molecular mechanisms underlie distinct regenerative responses, leading to blastema formation in animals and callus formation in plants. Both processes are driven by stem cell activation and rely on functionally analogous signaling pathways and regulatory molecules, including phytohormones in plants and GFs in animals, to initiate tissue repair [128]. Despite differences in cellular organization and developmental signaling, these regeneration pathways share core mechanisms, particularly the dedifferentiation of specialized cells into pluripotent or progenitor-like states [129]. The formation and maintenance of the blastema are tightly regulated by signaling pathways, including retinoic acid, FGF, and WNT/β-catenin signaling, which drive cell cycle re-entry and maintain the proliferative state of blastemal cells [100]. In contrast, the functional equivalent of a blastema in plants is the callus, a mass of dedifferentiated cells from which regenerated tissues can arise, contributing to their broader capacity for tissue regeneration across species [5]. In plants, callus formation is a common, though not universal, response to injury. A callus is typically a disorganized growth, arising on wound stumps and in response to certain pathogens [130]. The capacity of callus cells for trans-differentiation allows plants to regenerate virtually any tissue, providing the basis for their ability to be propagated from cuttings [10] (Table 1 and Figure 4).

Plant Stem Cells vs. Animal Stem Cells. Stem cells form the biological basis of regeneration in both plants and animals, playing essential roles in tissue repair, development, and cellular turnover. Regenerative processes are initiated by stem cells with high developmental potential, including totipotent cells capable of generating all cell types, which subsequently give rise to pluripotent and lineage-restricted stem cells that support tissue specialization and organismal growth. Despite profound evolutionary divergence, plant and animal stem cells share key functional properties, including self-renewal capacity and responsiveness to conserve regulatory signals at molecular and developmental levels. These similarities suggest the existence of a broadly conserved regenerative framework across biological kingdoms [23].

Plant Meristem vs. Animal Niche. In both plants and animals, the stem cell niche provides a dynamic microenvironment that ensures proper stem cell function [105]. In animals, stem cells are found in niches across various tissues, including hematopoietic, intestinal, skin, and neural tissues. These niches regulate stem cell behavior through signaling pathways such as WNT and Notch, essential for self-renewal and tissue regeneration [131]. In plants, stem cells are housed in specialized regions known as meristems, which are essential for post-embryonic development. Stem cells within these meristems are regulated by key transcription factors, such as WUSCHEL (WUS), and a ligand receptor signaling cascade called the CLAVATA (CLV) pathway, both crucial for maintaining pluripotency and stem cell homeostasis [132].

Molecular Pathways Regulating Stem Cells in Plants vs. Animals. While both plant and animal stem cells share the capacity for regeneration, the underlying molecular pathways show both similarities and distinctions. In animals, the retinoblastoma-related proteins (RBR) regulate stem cell activity by controlling differentiation and self-renewal, and this mechanism is also conserved in plants [133]. Additionally, the TOR pathway is evolutionarily conserved across plants and animals, playing an essential role in coordinating cell growth, division, and regeneration in response to external signals [134]. In animals, TOR integrates signals related to hormones, energy, and nutrition [135], while in plants it controls callus formation, stem growth, and root development [134].

Plant vs. Animal in Epigenetic Regulation and DNA Methylation. Epigenetic regulation plays a fundamental role in stem cell maintenance in both plants and animals. In animals, DNA methyltransferase 1 (DNMT1) preserves methylation patterns during DNA replication, ensuring genomic stability and regulating stem cell self-renewal and differentiation [136]. Similarly, plants utilize Methyltransferase 1 (MET1), a homolog of DNMT1, to maintain CG methylation and ensure stable gene expression and proper stem cell function [137].

Plant vs. Animal Antiviral Defense of Stem Cells. Both plant and animal stem cells have developed antiviral mechanisms to preserve their integrity. In human stem cells, the AviD is involved in defense against RNA viruses, a protective strategy similarly observed in plant stem cells [104].

Plant vs. Animal Vascularization. Vascular systems in both plants and animals perform the fundamental role of transporting vital substances such as nutrients, gases, and signaling molecules throughout the organism. However, the structure and developmental logic of these systems differ markedly between kingdoms. In animals, vascular tissues consist of hollow, non-cellular interior spaces lined by ECs, designed for rapid, pressurized blood flow. In contrast, plant vasculature is composed of living and dead cells arranged in continuous strands; xylem and phloem elements are derived from cellular systems [138]. One significant point of convergence between plants and animals, however, is the wound-responsive regeneration of vascular tissues. Both kingdoms exhibit mechanisms that are sensitive to wound size and age of the injured tissue. For instance, recent studies in Arabidopsis have reported, under specific experimental conditions, that vascular regeneration in leaves can bridge gaps of up to approximately 400 µm in the midvein. When the wound is below this threshold, new vascular strands can reconnect severed tissues or form alternate pathways to nearby lateral veins, effectively restoring functionality. However, larger disruptions exceeding this critical distance fail to regenerate, indicating a structural and developmental limit to tissue repair. Like vertebrate systems, age significantly impacts regenerative capacity. In animals, the decline in tissue regeneration with aging is well-documented, attributed to diminished stem cell activity and systemic changes in signaling [139]. In plants, leaf age likewise imposes limitations on vascular regeneration. Although mechanical wounding is more feasible in mature leaves, their capacity to regenerate vascular connections diminishes dramatically. Even when wound gaps remain within the permissible range, older leaves such as those at 10 days post-germination often fail to initiate or complete vascular repair [84].

Scar Formation in Plants vs. Animals. Scar formation represents a fundamental outcome of wound healing in animals and is often used descriptively in plant systems, although the underlying mechanisms differ substantially between the two kingdoms. In mammals, scar formation typically arises during the repair of extensive or deep injuries and reflects a default fibrotic response aimed at rapidly restoring tissue integrity. During this process, wound exudates provide GFs, nutrients, and immune cells that support tissue repair while maintaining a hydrated microenvironment [140]. The remodeling phase, which may begin approximately one week after injury and persist for several months, ultimately determines whether healing proceeds toward regeneration or fibrosis. Excessive or prolonged inflammatory and proliferative responses, such as those associated with infection or burns, significantly increase the likelihood of fibrotic scar formation [114]. In this context, scar formation is largely driven by collagen accumulation regulated by TGF-β signaling, which orchestrates ECM organization and promotes fibroblast proliferation and migration. Conversely, reduced infiltration of inflammatory cells, including mast cells, neutrophils, and macrophages, has been associated with attenuated scar formation, demonstrating the role of tightly controlled immune responses [115]. Notably, certain biological contexts such as pre-third-trimester fetal skin, the oral mucosa, and regenerative species like the African spiny mouse demonstrate scar-free healing, demonstrating that fibrosis is not an inevitable outcome but rather a regulated endpoint of tissue repair [99].

In contrast, scar formation in plants is mechanistically and structurally distinct from fibrotic scar formation in animals but remains functionally analogous in restoring tissue integrity, reflecting a fundamentally barrier-based repair strategy. Following injury, plants initiate a rapid wound response characterized by desiccation of exposed cells and activation of adjacent parenchyma cells, which undergo lignification and suberization to form a chemically reinforced boundary. In plant systems, the term “scar” is widely used particularly in fruits, leaves, and stems to describe visible structural alterations following injury. However, these structures arise from layered barrier-forming processes, including lignification, suberization, and wound periderm formation, which typically occur several cell layers inward rather than at the exposed wound surface and do not involve collagen-based fibrotic remodeling [141,142].

This initial sealing phase is followed by the formation of a wound periderm, a secondary protective tissue derived from the differentiation of a phellogen (cork cambium), which produces suberized phellem outward and phelloderm inward. The resulting ligno-suberized structure functions as a long-term protective barrier that restores tissue integrity without reconstructing the original cellular architecture [143,144,145]. Despite these mechanistic differences, scar formation in both systems fulfills a functionally analogous role: the rapid re-establishment of a protective interface between internal tissues and the external environment. In animals, this is achieved through collagen-rich fibrotic tissue, whereas in plants it relies on suberin- and lignin-based barrier formation. However, the consequences of scar formation extend beyond biological function to include important practical implications. In plants, particularly in agricultural systems, visible scarring on fruits such as tomato or tamarillo can significantly reduce marketability and economic value due to perceived defects [146]. Similarly, in humans and other animals, cutaneous scars may lead to aesthetic concerns and psychological distress, especially when located in exposed areas of the body [114]. Collectively, these observations demonstrate that although plant and animal scar formation arise from distinct structural frameworks suberin/lignin-based barriers versus collagen-based fibrosis they represent functionally analogous strategies that prioritize rapid protection over complete regeneration. This convergence underscores a fundamental principle in wound biology across kingdoms: when regeneration is limited or delayed, organisms favor the formation of a stable, protective barrier to ensure survival (Figure 4).

## 2. Insights into Regenerative Medicine Through Bioinspired Therapeutic Strategies

### 2.1. Comparative Insights for Regenerative Medicine

Chronic wounds, including diabetic foot, venous, and pressure ulcers, remain a major unmet medical challenge because of their multifactorial pathophysiology and the limited efficacy of current treatments [147]. Chronic non-healing lesions affect millions of patients worldwide, with 40–50% failing to heal after four weeks of standard care, thereby imposing a substantial healthcare burden [148]. Among the major factors contributing to this impaired healing process, hypoxia plays a central role. Hypoxia, often associated with ischemic tissues, disrupts cellular metabolism and alters inflammatory and reparative responses, thereby impairing tissue regeneration and contributing to persistent inflammation and delayed recovery [149]. Despite the availability of several therapeutic interventions, overcoming these pathological barriers remains challenging. Conventional approaches, including wound offloading, debridement, antibacterial agents, and hyperbaric oxygen therapy, often require repeated interventions and may not adequately address the multiple factors that sustain disease chronicity [150]. These wounds are inherently multifactorial conditions requiring simultaneous intervention across several chronicity-inducing factors, whereas current medical devices and pharmacological therapies frequently target only isolated aspects of the underlying pathology [147]. Moreover, oxygen delivery from current devices is typically short-lived, limiting long-term therapeutic efficacy. Therefore, there is an urgent need for therapeutic strategies capable of providing sustained oxygen delivery, infection control, and coordinated modulation of the complex biological processes involved in tissue repair and regeneration [151]. The applications and research are based on the differences in ulcer types, providing distinct therapeutic possibilities.

Among the regenerative strategies currently being explored, stem cell-based therapies have emerged as particularly promising approaches because of their ability to modulate inflammation, promote angiogenesis, and support tissue repair [152]. MSCs exhibit strong immunomodulatory activity and secrete bioactive mediators, including cytokines, GFs, and extracellular vesicles, which promote angiogenesis, regulate inflammation, and enhance tissue repair in both acute and chronic wounds [153]. MSC-based approaches have also been explored in skin substitutes and regenerative therapies [154]. Despite these advances, several limitations restrict their clinical translation, including immune rejection, tumorigenic risk, donor variability, and challenges in dosing and delivery. In addition, scalability constraints, high production costs, reduced efficacy after cryopreservation, and strict regulatory requirements continue to limit widespread clinical application in dermatology and wound care [153].

In addition to conventional stem cell-based therapies, plant stem cells and their derived products have attracted increasing interest as potential sources of bioinspired regenerative strategies. Plant stem cells possess several characteristics that make them particularly interesting for exploratory regenerative applications. They can be readily obtained from natural sources and expanded in vitro at relatively low cost, supporting scalability and sustainability. Importantly, plant-derived products are free from human pathogens and exhibit favorable safety profiles compared with many synthetic alternatives [155]. Beyond these practical advantages, plant stem cells produce diverse bioactive compounds, including antioxidants, and secondary metabolites, which can modulate oxidative stress, inflammation, and cellular proliferation, all of which are key processes in tissue repair [156]. Experimental studies have shown that plant stem cell-derived extracts may enhance skin renewal and support wound-healing-related processes [157].

These observations highlight the potential of plant stem cells and plant-derived products as sources of bioinspired regenerative strategies. However, despite this promise, no established therapeutic strategy based on the direct application of living plant stem cells to human wounds has yet been demonstrated. One of the major barriers to clinical translation is the environmental incompatibility between plant cells and human tissues, including differences in temperature, light requirements, ionic balance, and gas exchange [158]. Nevertheless, preliminary clinical evidence suggests that plant stem cell-derived products may provide therapeutic benefits. For example, Elboraey et al. (2023) reported the safe use of Edelweiss stem cell-derived products and extracts without observable adverse effects, indicating their potential suitability for regenerative applications [159]. Together, these findings highlight both the opportunities and the current limitations of living plant stem cells, emphasizing the need for innovative strategies that can support their viability and functionality within wound environments.

### 2.2. Hybrid-Wound Healing System: Concept and Biological Feasibility

Inspired by the comparative insights presented in this review, we propose the hybrid-wound healing system as a conceptual framework for exploring the potential application of living plant stem cells, including callus-derived and other regenerative plant cell culture, in wound therapy. Within this framework, living plant stem cells could, in theory, function as pharmaceutical microfactories capable of continuously producing and releasing bioactive compounds in response to the dynamic requirements of wound bed. This hypothesis emerged from observations that plant stem cells and regenerative plant cell culture systems can produce a wide range of bioactive compounds involved in stress adaptation, defense responses, and tissue regeneration. In addition, several studies have demonstrated that non-mammalian living biological systems, including photosynthetic microorganisms and engineered living biomaterials, can remain functional within controlled therapeutic platforms. These observations provide a conceptual basis for exploring whether regenerative plant-derived cells, particularly plant stem cells, could be incorporated into engineered wound-healing systems. However, their survival, functionality, and therapeutic relevance within human wound environments remain unproven and require substantial experimental validation.

**Figure 5:**
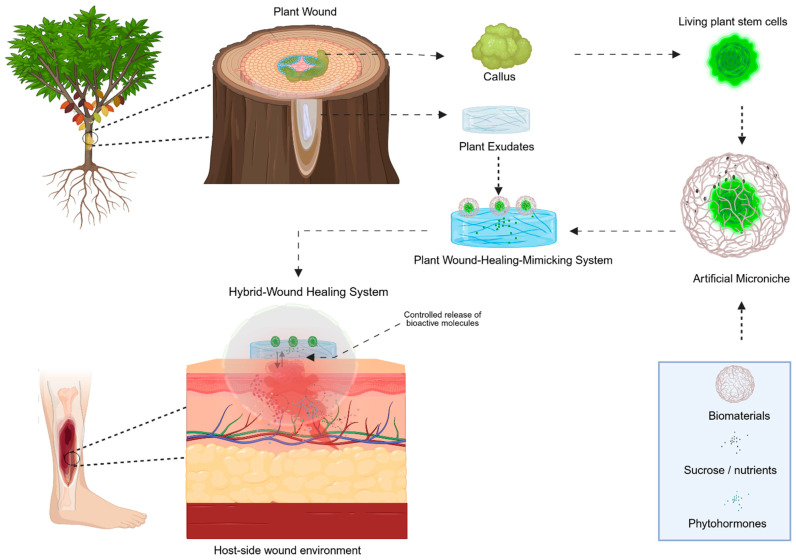
Proposed Hybrid-Wound Healing System as a conceptual framework for plant-inspired regenerative medicine. Living plant stem cells derived from wound-responsive plant tissues are maintained within an Artificial Microniche containing some biomaterials, nutrients, and phytohormones. Together with plant exudates, these components generate a Plant Wound-Healing-Mimicking System designed to reproduce selected biological characteristics of plant wound repair. The proposed system is conceptually completed upon interaction with the host-side wound environment, where plant-derived and animal wound-healing processes are hypothesized to function cooperatively. The resulting hybrid-wound healing system represents a hypothesis-generating framework inspired by shared regenerative mechanisms identified in plants and animals. Created in BioRender. Lima, F. (2026) https://BioRender.com/rhrgz6l, accessed on 18 June 2026.

So, the hybrid-wound healing system is proposed as a conceptual framework comprising two interconnected environments. The first environment is an Artificial Microniche containing living plant stem cells and a supportive microenvironment composed of biomaterials, nutrients, phytohormones, and other supportive factors intended to promote cell maintenance and metabolic activity. This compartment is intended to mimic selected features of plant stem-cell niches, such as meristematic tissues, and to provide a supportive microenvironment that may help reduce osmotic stress. The second environment comprises plant exudates and the host wound bed. Plant exudates serve as a biochemical interface between the Artificial Microniche and the host tissue. Together, the Artificial Microniche and plant exudates conceptually establish a Plant Wound-Healing-Mimicking System that reproduces selected biochemical characteristics of plant wound repair. Upon interaction with the host-side wound environment, this plant-inspired regenerative system forms the proposed hybrid-wound healing system, in which plant-derived and animal wound-healing processes are hypothesized to function cooperatively. The selection and formulation of plant exudates are guided by plant origin, exudate type, and target wound characteristics. This conceptual design was developed to address some of the biological and environmental challenges associated with the direct transplantation of plant stem cells into wound environments. Therefore, the proposed hybrid-wound healing system should be considered a hypothesis-generating framework intended to guide future research rather than an experimentally validated therapeutic platform (Figure 5).

Although the proposed hybrid-wound healing system remains a conceptual framework, several recent studies have demonstrated that living biological systems can be successfully incorporated into engineered wound-healing platforms. In particular, photosynthetic microecological hydrogels containing *Chlorella* and *Bacillus subtilis* have shown promising therapeutic effects in chronic wound models by simultaneously alleviating hypoxia, suppressing pathogenic microorganisms, and promoting tissue regeneration [148]. Likewise, photosynthetic microalgae can continuously generate oxygen and bioelectric signals, functions that have been associated with enhanced angiogenesis, collagen deposition, and re-epithelialization during wound repair [151]. Chlorophyll and related pigments further expand their therapeutic potential by acting as natural photosensitizers, enabling photodynamic therapy and antimicrobial effects [160]. Importantly, photosynthetic microalgae, bacterial systems, plant extracts, and photosynthetic biomaterials are biologically distinct from living plant stem cells and should not be considered equivalent regenerative platforms. Consequently, evidence obtained from these systems does not constitute direct evidence supporting the therapeutic application of living plant stem cells in wound environments. Rather, such studies provide conceptual precedents demonstrating that non-mammalian living biological systems can be maintained within engineered therapeutic platforms and may contribute beneficial functions under controlled conditions.

## 3. Limitations and Future Perspectives

Despite the promise of the concepts discussed in this review, several limitations should be acknowledged. As a narrative and descriptive rather than systematic review, the present work may not capture all relevant studies within the rapidly evolving fields of wound healing, regenerative medicine, and plant biology. In addition, comparative data directly linking plant and animal wound-healing processes remain limited, and the molecular basis underlying many of the proposed conserved responses has not yet been fully elucidated. Furthermore, many similar processes have phylogenetic, biomolecular, and physiological regulatory bases with distinct complexities.

Importantly, no direct experimental evidence currently demonstrates that living plant stem cells can survive, function, or provide therapeutic benefits within human wound environments. Therefore, the proposed hybrid-wound healing system should be regarded as a conceptual framework intended to generate new hypotheses rather than as an experimentally validated therapeutic platform. Several translational challenges must also be addressed before such concepts can be explored clinically, including plant cell viability under physiological conditions, environmental compatibility, immune interactions, biosafety considerations, and the development of appropriate regulatory frameworks. Furthermore, the long-term behavior of living plant-derived biological systems in mammalian tissues remains unknown.

Future studies should focus on experimental validation of the proposed concepts through in vitro, ex vivo and in vivo models. Particular attention should be given to evaluating plant cell viability, bioactive molecule secretion, cytotoxicity, antimicrobial activity, host–cell interactions, and biosafety. Advances in biomaterials, artificial microenvironments, and cell-encapsulation technologies may provide opportunities to overcome current limitations and further explore the potential of bioinspired regenerative strategies derived from comparative plant–animal wound-healing biology.

## 4. Conclusions

This review provides a comparative perspective on wound-healing processes in plants and animals and addresses two important challenges in the field: the lack of a broadly organized framework for plant wound healing and the absence of a systematic approach for cross-kingdom comparison, respecting the important differences in the complexity, embryogenesis, biology, and physiology of the processes. By organizing wound-healing responses into three functional phases, bioelectrical signaling, immune responses, and tissue formation and remodeling, we established a common framework that facilitates the systematic analysis of regenerative processes across evolutionarily distant organisms. Application of this framework revealed multiple shared cellular and molecular mechanisms involved in injury perception, defense activation, tissue regeneration, and remodeling. Despite substantial differences in anatomy and physiology, plants and animals employ similar biological strategies to restore tissue integrity, highlighting conserved regenerative principles that transcend kingdom boundaries despite the biomolecular and physiological differences. These findings demonstrate the value of comparative biology for identifying similar mechanisms that may otherwise remain overlooked when plant and animal systems are studied independently. The identification of these shared regenerative mechanisms also provides a foundation for the development of new bioinspired concepts in regenerative medicine. In this context, the proposed hybrid-wound healing system should be viewed as a conceptual outcome emerging from comparative analysis rather than as an established therapeutic strategy. By integrating insights into plant biology, wound healing, and regenerative medicine, this work offers a framework for future interdisciplinary research and contributes to a broader understanding of regeneration across biological kingdoms.

## Figures and Tables

**Table 1:** Comparative analysis of major wound-healing processes in plants and animals. Similarities are categorized as established similarities (ES), functional similarities (FS), or functional analogies (FA), according to the level of experimental evidence and the nature of the biological relationship.

Wound Steps	Animal	Plant	Comparison Type
1. Bioelectrical signaling	GLU	GLU	ES
	Calcium waves	Calcium waves	ES
	Hydraulic waves (blood leakage)	Hydraulic waves (phloem release)	FS
	ROS waves	ROS waves	FS
	Temperature	Temperature	FA
2. Immune Responses	PG	JA	FA
	ABA	ABA	ES
	Melatonin	Melatonin	ES
	Animal exudates	Plant exudates (latex, resin, other)	FA
	Antimicrobial peptides	Antimicrobial peptides	ES
3. Tissue Formation and Remodeling	Animal ECM	Plant ECM (cell wall-associated extracellular compartment)	FA
	Blastema	Callus	FA
	Animal Stem Cells	Plant Stem Cells	FA
	Stem cell niche	Meristem	FA
	Wnt	WUS	FA
	Notch	CLV	FA
	RBR	RBR	ES
	TOR	TOR	ES
	*DNMT1*	*MET1*	FS
	Animal vascularization	Plant vascularization	FS
	Scar formation	Scar formation	FA

**Note:** Established Similarit (ES): Supported by substantial experimental evidence in both plant and animal systems.Functional Similarity (FS): Similar biological functions are observed in both kingdoms, although the underlying mechanisms, structures, or pathways may differ. Functional Analogy (FA): Conceptually comparable biological processes that perform similar functions but should not be interpreted as biologically identical structures, pathways, or developmental programs. Abbreviations: GLU, glutamate; ROS, reactive oxygen species; ECM, extracellular matrix; ABA, abscisic acid; TOR, target of rapamycin; WUS, WUSCHEL; CLV, CLAVATA; RBR, Retinoblastoma-related; *DNMT1*, DNA methyltransferase 1; *MET1*, Methyltransferase 1; ES, Established Similarity; FS, Functional Similarity; FA, Functional Analogy.

## Data Availability

No new data were created or analyzed in this study. Data sharing is not applicable to this article.

## Abbreviations

The following abbreviations are used in this manuscript:

ABA	abscisic acid
AEC	apical epidermal cap
AMPs	antimicrobial peptides
AviD	antiviral Dicer
CLV	CLAVATA
DAMPs	Damage-Associated Molecular Patterns
DNMT1	DNA methyltransferase 1
ECM	extracellular matrix
ECs	endothelial cells
EGFR	epidermal growth factor receptor
EPCs	endothelial progenitor cells
EPSCs	epidermal stem cells
ES	Established Similarity
FA	Functional Analogy
FGF	fibroblast growth factor
FS	Functional Similarity
GFs	growth factors
GI	gastrointestinal
GLR	glutamate receptor-like
GLU	glutamate
hBD-2	human β-defensin 2
IL-1	Interleukin-1
IL-10	Interleukin-10
IL-6	Interleukin-6
IL-8	Interleukin-8
JA	jasmonic acid
LL37	human cathelicidin antimicrobial peptide LL37
LPS	lipopolysaccharides
MMPs	matrix metalloproteinases
MSCs	mesenchymal stem cells
NO	nitric oxide
PAMPs	pathogen-associated molecular patterns
PDGF	platelet-derived growth factor
PGE2	prostaglandin E2
PGs	prostaglandins
RBbR	retinoblastoma-related proteins
REF1	regeneration factor 1
RNA	ribonucleic acid
RNS	reactive nitrogen species
ROS	reactive oxygen species
SEs	sieve elements
TGF-β	transforming growth factor-beta
TIMPs	tissue inhibitors of metalloproteinases
TNF	tumor necrosis factor
TOR	target of rapamycin
VEGF	vascular endothelial growth factor
WNT	Wingless-related integration site signaling pathway
WUS	WUSCHEL
